# Timing of adequate antibiotic therapy is a greater determinant of outcome than are TNF and IL-10 polymorphisms in patients with sepsis

**DOI:** 10.1186/cc4995

**Published:** 2006-07-19

**Authors:** Jose Garnacho-Montero, Teresa Aldabo-Pallas, Carmen Garnacho-Montero, Aurelio Cayuela, Rocio Jiménez, Sonia Barroso, Carlos Ortiz-Leyba

**Affiliations:** 1 Intensive Care Unit, Hospital Universitatrio Virgen del Rocio, Seviilla, Spain; 2 Institute for Environmental Medicine, University of Pennsylvania, Philadelphia, Pennsylvania, USA; 3 Supportive Research Unit, Hospital Universitario Virgen del Rocio, Sevialla, Spain

## Abstract

**Introduction:**

Genetic variations may influence clinical outcomes in patients with sepsis. The present study was conducted to evaluate the impact on mortality of three polymorphisms after adjusting for confounding variables, and to assess the factors involved in progression of the inflammatory response in septic patients.

**Method:**

The inception cohort study included all Caucasian adults admitted to the hospital with sepsis. Sepsis severity, microbiological information and clinical variables were recorded. Three polymorphisms were identified in all patients by PCR: the tumour necrosis factor (TNF)-α 308 promoter polymorphism; the polymorphism in the first intron of the TNF-β gene; and the IL-10-1082 promoter polymorphism. Patients included in the study were followed up for 90 days after hospital admission.

**Results:**

A group of 224 patients was enrolled in the present study. We did not find a significant association among any of the three polymorphisms and mortality or worsening inflammatory response. By multivariate logistic regression analysis, only two factors were independently associated with mortality, namely Acute Physiology and Chronic Health Evaluation (APACHE) II score and delayed initiation of adequate antibiotic therapy. In septic shock patients (*n *= 114), the delay in initiation of adequate antibiotic therapy was the only independent predictor of mortality. Risk factors for impairment in inflammatory response were APACHE II score, positive blood culture and delayed initiation of adequate antibiotic therapy.

**Conclusion:**

This study emphasizes that prompt and adequate antibiotic therapy is the cornerstone of therapy in sepsis. The three polymorphisms evaluated in the present study appear not to influence the outcome of patients admitted to the hospital with sepsis.

## Introduction

Mortality from sepsis remains unacceptably high despite recent advances in diagnostic procedures, antimicrobial treatment, and supportive care [1,2]. Although antibiotic therapy is the cornerstone of treatment of infections, the influence of adequate antimicrobial therapy on prognosis in septic patients was not clearly proven until recently. We and others demonstrated that after controlling for confounding variables, adequate empirical antibiotic treatment is associated with reduced mortality in critically ill septic patients [3-5]. Interestingly, the impact on outcome of delayed adequate antibiotic therapy has not been studied in septic patients. The variables that can influence outcome in septic patients are numerous (such as, age, underlying disease, source of sepsis, presence of bacteraemia and organ system dysfunction). In addition, several interventions have been shown to decrease mortality in sepsis and should be taken into account when analyzing the impact on outcome of any single factor.

At the beginning of the third millennium, much interest and great expectation were focused on advances in molecular biology, and particularly on the completion of Human Genome Project. Individual variants of genes encoding mediators that are involved in the inflammatory response to an infectious agent might account for differences in clinical evolution and outcome of septic patients treated correctly and with apparently similar approaches. Tumour necrosis factor (TNF) is considered the most important proinflammatory cytokine, recruiting and activating immune cells, stimulating the release of other proinflammatory mediators and regulating apoptosis. In contrast, IL-10 is the paradigmatic anti-inflammatory cytokine. It exerts its biological properties by inhibiting the release of proinflammatory cytokines and preventing apoptosis. However, contradictory observations have been reported on the impact on outcome of polymorphisms in these cytokines that are directly responsible for the host response [6,7].

An international panel of experts defined systemic inflammatory response syndrome, sepsis, severe sepsis and septic shock, which are viewed as a continuum of risk [8]. Factors that influence the progression of inflammatory response in patients admitted to the hospital because of sepsis have not been elucidated. In other words, the causes why clinical situation deteriorates are not clearly known.

Hence, the primary aim of the present study, conducted in adults admitted to the hospital with sepsis, was to evaluate the impact on in-hospital mortality (and 90-day mortality) of three polymorphisms (the TNF-α-308 promoter polymorphism, the polymorphism in the first intron of the TNF-β gene, and the IL-10-1082 promoter polymorphism), after controlling for various confounding variables including delayed adequate antibiotic therapy. We chose these mediators because an altered balance in their serum concentrations has been associated with poor outcome in patients with community-acquired infection [9], and we selected these specific polymorphisms because of their functional significance [10-12]. Our secondary objectives were to explore this same objective in patients with septic shock and to assess the factors that are involved in progression of the inflammatory response.

## Materials and methods

### Hospital

This prospective study was carried out in the Hospital Virgen del Rocío – a large university hospital with a 40-bed intensive care unit (ICU) – from June 2002 to December 2004. Written informed consent was obtained from patients or their relatives, and the ethics committee of the hospital approved the study.

### Patients

Eligible patients were Caucasian adults (age >18 years) who arrived in at the emergency department meeting criteria for sepsis. Infected patients who did not fulfill sepsis criteria were not included. The criteria followed for ICU admission were based on the patient clinical condition, being shock or respiratory insufficiency the main reasons for ICU admission. Patients not admitted to the ICU were transferred to the general ward. The exclusion criteria were neutropenia (white blood cell count <500/μl), positive HIV serologic results and pregnancy. Patients included in the protocol were followed up until death or hospital discharge. Vital status of those patients discharged from the hospital before 90 days was ascertained by searching in the hospital database or telephone contact. The control group comprised 101 healthy unrelated blood donors from the hospital blood bank.

### Study design

All patients diagnosed with sepsis received standard supportive treatment, including prompt fluid resuscitation, vasoactive drugs and empirical antimicrobial therapy, which was chosen by the attending physician [3]. Intravenous hydrocortisone (200 mg/day for seven days) was used in patients in septic shock who, despite fluid replacement, required vasopressor agents [13], and continuous infusion of insulin was administered to control blood glucose levels, in accordance with the results of a large clinical trial [14]. All patients on mechanical ventilation were managed following ARDSNet recommendations [15].

All patients had a series of blood cultures drawn at admission. Sepsis was documented when a relevant micro-organism from a suspected focus was isolated and/or bacteraemia was present. Cultures of infection sources were obtained in all patients as clinically indicated. Paired serum samples were tested for evidence of antibody against respiratory viruses, *Legionella pneumophila*, *Chlamydia *spp., *Coxiella burnetii *and *Mycoplasma pneumoniae*. Tracheal aspirate or protected brush specimen were obtained from all patients with community-acquired pneumonia who required mechanical ventilation.

### Variables

Sepsis, severe sepsis and septic shock were defined following current definitions [8]. Severity of illness was evaluated using the Acute Physiology and Chronic Health Evaluation (APACHE) II score, recording the worst reading during the first 24 hours in the hospital [16]. Chronic organ insufficiencies (liver, renal, pulmonary, cardiovascular and immunosuppression) were recorded as defined in the APACHE II scale, and other comorbidities (alcoholism, smoking habit, diabetes mellitus and noncured malignancy) as defined by Pittet and coworkers [17]. Other variables recorded included bacteraemia, microbiologically documented infection and delay from hospital admission (documented time when the patient arrived at the emergency department) to administration of adequate antibiotic therapy. Time elapsed from hospital admission to onset of operation was noted in surgical patients. Empirical therapy was considered adequate when at least one effective drug was included in the antibiotic treatment regimen within the first 24 hours in the hospital, and the dose and pattern of administration were in accordance with current standards.

Failure of organs was evaluated using the Sequential Organ Failure Assessment (SOFA) scale on admission and during the subsequent clinical course [18]. Worsening in the inflammatory response was monitored by two methods: we determined the proportion of patients admitted to the hospital with sepsis who developed severe sepsis or septic shock and the proportion of patients with severe sepsis at admission who developed septic shock; and we calculated the delta-SOFA (i.e. the worst SOFA score during hospitalization minus the SOFA score during the first 24 hours) [19]. Nosocomial infections (pneumonia, catheter-related bloodstream infection and primary bacteraemia) were diagnosed as previously defined [20].

### Genotyping

Genomic DNA from each patient was extracted from whole blood using a DNA extraction Kit (Puregene DNA Isolation kit, Minneapolis, MN, USA), in accordance with the manufacturer's instructions.

#### Polymorphism at TNF-α promoter position -308

DNA samples were amplified by PCR with forward primer TNF-α-F (5'-AGG CAA TAG GTT TTG AGG GCC AT-3') and reverse primer TNF-α-R (5'-ACA CTC CCC ATC CTC CCT GCT-3'). The TNF-α primer includes a single base pair (bp) mismatch, which introduces an *Nco*I restriction site after amplification when the G allele is present at position -308. A 116 bp PCR product was obtained from a 50 μl reaction mix containing 100 ng DNA, 1 μmol/l each primer, 0.2 mmol/l dNTP, 1× buffer, 1.5 mmol/l MgCl_2 _and 1 U Taq polymerase (Finnzymes, Espoo, Finland). The reaction was carried out with the following cycles: 95°C for 2 minutes; 35 cycles of 95°C for 30 s, 60°C for 15 s and 74°C for 15 s; and 74°C for 10 minutes for final extension. The PCR product was incubated with *Nco*I (New England Biolabs) and digested, and undigested samples were visualized by electrophoresis in 4% Nu Sieve agarose gel (Master Diagnostica, Madrid, Spain) and ethidium bromide staining. A single band at 116 bp identified AA homozygous individuals, two bands at 96 and 20 bp identified GG homozygous individuals, and three bands at 116, 96 and 20 bp indicated a heterozygote at the TNF-α-308 locus.

#### Polymorphism in the first intron of the TNF-β gene (NcoI polymorphism)

The region with the +250 polymorphism, which contains an *Nco*I restriction site when the G allele is present, was amplified using primers TNF-β-F (5'-CCG TGC TTC GTG CTT TGG ACT A-3') and TNF-β-R (5'-AGA GGG GTG GAT GCT TGG GTT C-3'). Each 50 μl reaction mix consisted of 100 ng DNA, 1 μmol/l each primer, 0.2 mmol/l dNTP, 1× buffer, 1.5 mmol/l MgCl_2 _and 1 U Taq polymerase. Amplification was performed with an initial denaturation of 95°C for 2 minutes; followed by 35 cycles of 94°C for 30 s, 69°C for 30 s and 74°C for 42 s; and completing the reaction with a final extension step of 74°C for 10 minutes. The 782 bp PCR product was digested with the restriction enzyme *Nco*I (New England Biolabs), incubating at 37°C for two hours. The obtained fragments as well as undigested samples were analyzed by electrophoresis in 1.5% agarose gel and visualized by ethidium bromide staining. The presence of a single band of 782 bp identified individuals who were AA homozygous, two bands at 586 and 196 bp indicated those who were GG homozygous, and heterozygous individuals were identified by three bands at 782, 586 and 196 bp.

#### Polymorphism at IL-10 promoter position -1082

IL-10-1082 polymorphism results from the substitution of guanine with adenine, which abolishes a *Mnl*I restriction site. The region containing this polymorphism was amplified by PCR using primers IL-10-F (5'-CTC GTC GCA ACC CAA CTG-3') and IL-10-R (5'-ACT TTC ATC TTA CCT ATC CCT ACT TCC-3'). The amplified region also includes the CA repeat microsatellites located at -1151 (IL-10-G polymorphism). A 50 μl reaction mix contained 100 ng DNA, 0.5 μmol/l each primer, 0.2 mmol/l dNTP, 1× buffer, 1.5 mmol/l MgCl_2 _and 1 U Taq polymerase. The PCR conditions used were as follows: a denaturing step of 94°C for 3 minutes and then 35 cycles of 94°C for 15 s, 60°C for 15 s and 72°C for 30 s, with a final extension at 72°C for five minutes. The PCR product was analyzed by electrophoresis in 4% Nu Sieve agarose gel (Master Diagnostica) and visualized by ethidium bromide staining. An undigested single band at 139 bp (when 20 CA repeats are present) identified individuals who were AA homozygous, two bands at 101 and 38 bp identified those who were GG homozygous, and three bands at 139, 101 and 38 bp indicated a heterozygote at the -1082 locus.

### Statistical analysis

The main outcome measure was in-hospital mortality from any cause, but we also assessed 90-day mortality. Sample size was calculated considering a difference of 10% in hospital mortality between groups as relevant, the frequency of the alleles in the population (control group), and a β error of 20% and an α error of 5%. An interim analysis was planned after 225 patients were enrolled. At that point, the absolute difference in mortality between AA individuals of TNF-β (the polymorphisms more consistently associated with mortality) and GG/GA individuals was only 5%, and this was considered not clinically relevant in septic patients, whose outcome is influenced by many variables. With these data, it would be required to enroll 2,500 patients to achieve statistical significance. For these reasons, we elected to terminate the study.

Descriptive results of continuous variables are expressed as median (25th and 75th percentiles). The association between risk factors and death was first examined by means of bivariate analysis. This was accomplished using two-sample unpaired t-test for continuous variables after correction for equality of variance (Levene's test), χ^2 ^test, or Fisher's exact test for categorical variables. Relative risks and their corresponding 95% confidence intervals (CIs) were calculated. *P *< 0.05 was considered to reflect statistical significance.

A stratified analysis was performed before the multivariate analysis using the Mantel-Hanszel χ^2 ^test, in order to evaluate the presence of interactions and confounding factors among variables. A multivariate analysis using logistic regression analysis was used to determine variables independently associated with mortality in the entire group and only in those patients who fulfilled septic shock criteria. The model was constructed using a forward stepwise method with the likelihood ratio test. The variables tested for inclusion in the model had an entry level *P *< 0.10 in the univariate analysis, but only those with *P *< 0.05 are reported. The odds ratios (ORs) and their corresponding 95% CI for each variable were also calculated [21]. Moreover, in order to assess the associations among quantitative variables with delayed initiation of adequate antibiotic therapy, a multiple linear regression analysis was performed.

## Results

A total of 293 patients were screened for inclusion in the study, although 69 were excluded from the final analysis. Therefore, only 224 patients were evaluable. The causes of exclusion are listed in Table 1. Forty-six patients were hospitalized in the general ward and 178 patients were admitted to the ICU (four of them were initially transferred to the general ward). Fifty-two patients died in the hospital (23.2%), whereas in-hospital mortality of the 69 excluded patients was 21.7% (*P *> 0.05). The demographic data and the distribution of genotypes of the 224 patients and 101 control individuals are summarized in Table 2. The distribution of genotypes for the analyzed polymorphisms did not differ between patients with sepsis and control individuals.

At admission to the hospital, a clinical picture of sepsis was present in 78 cases (34.8%), severe sepsis in 85 (38%) and septic shock in 61 (27.2%). Twenty patients who were admitted with sepsis developed severe sepsis or septic shock, and 37 patients with severe sepsis developed septic shock. Therefore, 58 patients fulfilled only sepsis criteria, 52 patients presented with severe sepsis criteria and 114 patients presented with septic shock. Only 10 patients received activated protein C as part of their treatment.

All patients had clinical signs of infection but the causal micro-organism could not be microbiologically documented in 66 patients (29.4%). Thirty-one patients presented with positive blood culture and focus of infection, 25 were only bacteraemic, and in 102 only culture of material from the apparent focus of infection was positive. Polymicrobial sepsis was diagnosed in 22 cases. Table 3 shows the micro-organisms isolated in blood and the sites of infection.

Empirical antibiotic therapy was inadequate in 16 patients (in-hospital mortality 75%). The reasons for inadequacy of antibiotic therapy were as follows: pathogen resistant to prescribed antibiotic (eight cases), pathogen not covered by empirical antibiotic therapy (four cases) and no antimicrobial administered within the first 24 hours in the hospital (four cases). In one case (bacteraemia of undetermined source caused by *Prevotella oris*) the patient died before they received adequate antimicrobial treatment.

### Predictors of in-hospital mortality

A bivariate analysis of risk factors for in-hospital mortality is reported in Table 4. The mortality rate was significantly higher for females than for males. Neither microbiological documentation of sepsis nor the presence of bacteraemia was related to a worse outcome. Median delay of initiation of adequate antibiotic therapy was significantly longer in nonsurvivors than in survivors (the patient who died before receiving adequate antimicrobial treatment was excluded from this analysis).

All genotype frequencies were in Hardy-Weinberg equilibrium. There were no significant differences in genotype or allele frequencies between survivors and nonsurvivors. A strong association was found between the TNF-α-308 and TNF-β (*Nco*I) polymorphisms. For TNF-α-308, 186 patients were GG homozygous and 135 of them (72.5%) were AA homozygous for the TNF-β *Nco*I polymorphism. In addition, we also assessed the impact on the outcome of the combination formed by GA/AA at position -308, AA homozygosity for TNF-β (*Nco*I polymorphism) and IL-10-1082 GG homozygosity, because this genotype could be considered the worst of the possibilities.

Twenty-seven episodes of nosocomial infection were diagnosed in 22 patients. Empirical antimicrobial therapy for the episode of nosocomial infection was inadequate in seven cases (26%). Mortality did not significantly differ between patients with (8/14 [36.4%]) and without nosocomial infection (44/158 [21.8%]; *P *= 0.12).

By multivariate logistic regression analysis, only two factors were independently associated with in-hospital mortality: APACHE II score (OR 1.18, 95% CI 1.04–1.35) and delayed initiation of adequate antibiotic therapy (OR 1.09, 95% CI 1.04–1.14).

### Predictors of 90-day mortality

Only 222 patients could be evaluated for 90-day mortality. Mortality rate was 25.7%. Multivariate analysis of risk factors for 90-day mortality was identical to the analysis of in-hospital mortality, with small differences in the OR.

### Patients with septic shock

We also explored the impact of analyzed factors and confounding variables on mortality in 114 patients with septic shock. Microbiological documentation of sepsis was achieved in 85 patients (74.6%). Bivariate analysis of risk factors for in-hospital mortality is reported in Table 5. No significant differences in genotype distribution or allele frequencies were found between survivors and nonsurvivors. The genotype formed by the combination of the allele A at position -308, AA homozygosity for TNF-β (Nco*I *Ncolpolymorphism) and IL-10-1082 GG homozygosity was not associated with a greater in-hospital mortality.

Twenty-two episodes of nosocomial infection were diagnosed in 18 patients (inadequate empirical antimicrobial therapy in five cases). Mortality rate was not statistically different in patients with or without nosocomial infection (50% versus 41.6%). Multivariate analysis confirmed that delayed adequate antibiotic therapy was the only independent predictor of in-hospital mortality (OR 1.06, 95% CI 1.01–1.10).

### Impairment in inflammatory response

The risk for impairment in the inflammatory condition was evaluated by comparing those 57 patients who exhibited a deterioration in inflammatory status (20 patients admitted with sepsis developed severe sepsis or septic shock and 37 patients with severe sepsis developed septic shock) with those 162 in whom the inflammatory response did not progress (61 patients were admitted to the hospital with the diagnosis of septic shock and were excluded from this analysis). The rate of impairment in inflammatory response was significantly greater in patients with inadequate empirical antibiotic therapy than in patients with adequate empirical antibiotic therapy (13/16 versus 34/142; *P *< 0.001). Bivariate analysis of risk factors for impairment in the inflammatory response is shown in Table 6. Multivariate analysis identified three variables independently associated with impairment of the inflammatory response: APACHE II score (OR 1.17, 95% CI 1.05–1.29), positive blood culture (OR 2.8, 95% CI 1.05–7.7), and delayed initiation of adequate antibiotic therapy (OR 1.14, 95% CI 1.06–1.24).

SOFA score increased in 111 patients (median increase in score 3 [range 1–6]). This increase was significantly greater in nonsurvivors (5 [2–8.75]) than in survivors (0 [0–1.75]; *P *< 0001). All patients with inadequate empirical antibiotic therapy within the first 24 hours of admission exhibited an increase in SOFA score.

**Figure 1:**
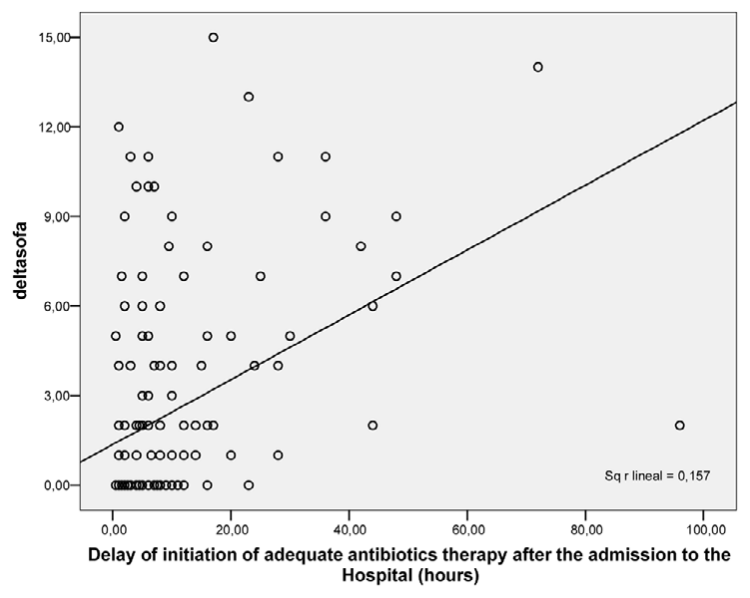
Correlation between delta-SOFA and delayed initiation of adequate antibiotic therapy. SOFA, Sequential Organ Failure Assessment.

Delta-SOFA correlated significantly with delayed initiation of adequate antibiotic therapy (*P *< 0.0001; Figure 1). By multiple linear regression, the only independent predictor of an increase in SOFA score was delayed initiation of adequate antibiotic therapy (*P *< 0.05).

## Discussion

The major finding of the present study is the importance of prompt and adequate antibiotic therapy on admission to hospital in patients with sepsis. Timely adequate antibiotic administration is associated with decreased mortality and a reduction in the impairment of inflammatory response, whereas there are no strong associations between selected TNF and IL-10 polymorphisms and outcomes.

The main purpose of identifying factors independently associated with mortality among septic patients is to recognize those variables associated with high risk for death. From a practical point of view, identified factors should be modifiable with medical interventions or be incorporated into our therapeutic arsenal if they are to achieve a reduction in mortality. The impact on outcome of early antibiotic treatment has been demonstrated for infections such as community-acquired pneumonia [22], although in patients with community-acquired meningitis there was a trend only in those who were less severely ill [23]. Interestingly, the impact of early adequate antibiotic therapy in patients with sepsis has never been assessed.

By multivariate analysis, we found that the risk for in-hospital mortality increased by 9% for every hour of delay to administration of the correct antibiotic regimen. Moreover, in patients with septic shock, the only independent predictor of in-hospital mortality was delayed administration of adequate antibiotic therapy. This finding is of great importance because septic shock is associated with high mortality rates, and the association of septic shock with death persists after adjusting for prognostic factors such as organ failure [2]. Valles and coworkers [4] found that inadequate antibiotic therapy was the most important determinant of survival in bacteraemic patients, and this finding was even more marked in patients with septic shock.

In the present study, mortality rates of septic patients with and without nosocomial infection were similar, although the incidence of sepsis in our study was lower than that in other series [24]. It should not be overlooked that one-fifth of our patients were not admitted to the ICU, we only noted severe nosocomial infections (urinary and wound infections were not noted), and community-acquired infection is a protective factor with respect to nosocomial infection in the ICU [25]. In any case, the impact on outcome of hospital-acquired infection seems not to be crucial in patients with sepsis at admission, and mortality is more directly related to the severity of illness and the initial management.

Approximately half of the patients presenting with sepsis deteriorated to a more severe stage in the inflammatory response during subsequent days [26]. In a recent multicentre study, Alberti and coworkers [27] proposed a score to forecast which patients may present with a deteriorating clinical state. In the present study, using two different approaches, delayed initiation of adequate antibiotic therapy was an independent predictor of impairment in inflammatory response. This is of the utmost importance because the more severe the inflammatory response, the greater the mortality rate.

Genetic variations within the TNF and IL-10 genes may influence mortality rates in patients with sepsis. Polymorphisms in these genes may determine the concentrations of proinflammatory and anti-inflammatory cytokines, and may influence whether patients have a marked hyper-inflammatory or anti-inflammatory response to infection. A delicate balance between inflammation and anti-inflammation is required if the adverse effects associated with predominance of either state is to be avoided. In sepsis, that elevated IL-10 serum levels have been associated with poor outcome might be a result of the development of immunoparalysis and increased risk for multiple organ dysfunction syndrome [9].

Previous studies have yielded conflicting findings on the impact on outcome of the two TNF polymorphisms in septic patients. In the case of the -308 (G/A) polymorphism, the frequency of TNF-2 (containing 'A') was higher among those septic shock patients who died, and this genotype was an independent predictor of mortality on multivariate analysis [10]. This association was also found by other investigators [28], but others were unable to demonstrate an association between this polymorphism and outcome in patients with sepsis [11]. Similar contradictory findings have been reported in the case of TNF-β (*Nco*I polymorphism); mortality in severe sepsis was higher in TNFB2 (AA) individuals [29,30], although other investigations found no such association [31]. A recent study enrolling 213 patients with severe sepsis [32] found no association between these polymorphisms and mortality. In our series, 72.5% of those who were GG homozygous for TNF-α-308 were AA homozygous for TNF-β' polymorphism, which is in agreement with a recent study that found these two polymorphisms to be in strong linkage disequilibrium [33].

Two studies conducted in patients with community-acquired pneumonia [34,35] found no association between the two TNF polymorphisms or the IL-10-1082 polymorphism and risk for developing septic shock or mortality. Moreover, patients with invasive pneumococcal disease who were GG homozygous for the IL-10-1082 polymorphism exhibited greater risk for septic shock, whereas mortality was unaffected [36]. In this study, the two TNF polymorphisms were not associated with a worse evolution of disease.

There is currently extensive disagreement on the value of association studies for the detection of genetic variants that contribute to death, especially in complex situations such as sepsis. Diverse methodological pitfalls may account for many contradictory results. Moreover, it must be acknowledged that the biological role of many of these polymorphisms remains to be elucidated. Thus, recent functional studies suggest that the much studied 308 G/A polymorphism is not functional, whereas the function of other TNF polymorphism remains controversial [37].

We acknowledge several limitations of our study. First, although a protocol following current recommendations for treatment of sepsis and septic shock was used (intravenous corticosteroids, continuous infusion of insulin and mechanical ventilation with low tidal volumes), we did not control certain variables that could have influenced outcomes, such as the total amount of fluid infused in the first few hours [38]. Second, the use of recombinant activated protein C in our study was very restricted, although this is not unusual in clinical practice [39]. Third, only three polymorphisms of two mediators were evaluated. Given the vast number of mediators that have been implicated in the response to an infectious agent, we cannot exclude the possibility that other polymorphisms not genotyped in the present study or specific haplotypes could influence survival [40]. Finally, our study may be underpowered to detect statistically significant differences, especially in septic shock patients, given the small sample size.

Despite these limitations, the study is unique in that it included an ethnically homogeneous population of patients admitted to the hospital with a diagnosis of sepsis, and evaluated the progression of the syndrome and the factors associated with mortality following their admission to the hospital. Among the factors analyzed, all enrolled patients were genotyped for three polymorphisms of two key mediators, following recent recommendations for genetic association studies [41]. Our findings emphasize that better use of conventional treatments is necessary before sophisticated interventions should be introduced into the clinical setting; we were unable to detect strong associations between these three polymorphisms and mortality in patients with sepsis at admission to hospital [42].

## Conclusion

Physicians should strive to initiate adequate antibiotic therapy as soon as possible in patients with sepsis. Clearly, surgical removal of the septic focus remains at the foundation of successful therapy, as is appropriate and prompt resuscitation of patients with severe sepsis and septic shock. Findings relating to the value of the association study, designed for detection of genetic variants contributing to death in septic patients, have been contradictory; this has resulted in increasing uncertainty among investigators and care givers. Therefore, large genetic studies are needed before genotypes can be incorporated into patient-tailored therapy. In the meantime, all educational programmes designed to reduce mortality in sepsis should specifically promote the correct and early use of antibiotics in different clinical settings.

## Key messages

• Early initiation of adequate antimicrobial therapy is life saving in patients admitted to hospital with sepsis.

• Timely and adequate antibiotic administration is associated with decreased mortality in patients admitted to the hospital meeting criteria for septic shock.

• The three polymorphisms evaluated in the present study (the TNF-α-308 promoter polymorphism, the polymorphism in the first intron of the TNF-β gene, and the IL-10-1082 promoter polymorphism) appear not to influence outcome in patients admitted to the hospital with sepsis.

• Delayed initiation of adequate antibiotic therapy is the only modifiable risk factor for deteriorating clinical condition, as assessed by delta-SOFA.

## Abbreviations

APACHE = Acute Physiology and Chronic Health Evaluation; bp = base pair; CI = confidence interval; ICU = intensive care unit; IL = interleukin; OR = odds ratio; PCR = polymerase chain reaction; SOFA = Sequential Organ Failure Assessment; TNF = tumour necrosis factor.

## Competing interests

The authors declare that they have no competing interests.

## Authors' contributions

JGM participated in the design of the study, ran the study, interpreted data, drafted the manuscript and obtained sponsorship. TAP participated in the acquisition, analysis and interpretation of data. CGM participated in the design of the study, carried out DNA testing and participated in the writing of the manuscript. RJ and SB participated in the acquisition of the data and performed DNA testing. AC conducted data analyses and interpreted the results. COL participated in the study design, acquisition of data and critical revision of the report. All authors read and approved the final manuscript.

## Figures and Tables

**Table 1:** Reasons for exclusion of 69 patients screened in the present study

Reason	Number
Permission denied	20
Diagnosis other than sepsis	20
Race other than Caucasian	13
Do not resuscitate order	8
Technical problem to extract DNA	2
Death before permission could be obtained	3
Other	3

**Table 2:** Demographic data and genotype frequencies of TNF and IL-10 polymorphisms in patients with sepsis and controls.

	Patients (*n *= 224)	Control individuals (*n *= 101)
Age	63.5 (49–72.75)	50 (46.5–55.5)
APACHE II	14 (9–19)	-
SOFA (1)^a^	4 (2–9)	-
-308 TNF-α promoter polymorphism		
GG	186 (83)	82 (81.2)
GA	35 (15.6)	15 (14.9)
AA	3 (1.4)	4 (3.9)
TNF-β (*Nco*I polymorphism)		
GG	16 (7.1)	10 (9.9)
GA	69 (30.8)	34 (33.7)
AA	139 (62.5)	57 (56.4)
IL-10-1082		
GG	33 (14.8)	15 (14.8)
GA	99 (44.2)	50 (49.5)
AA	92 (41)	36 (35.7)

Values are expressed as median (25th to 75th percentile) or as *n *(%).^a^SOFA (1) means SOFA score of the first 24 hours in the hospital. APACHE, Acute Physiology and Chronic Health Evaluation score; SOFA, Sequential Organ Failure Assessment; TNF, tumour necrosis factor.

**Table 3:** Micro-organisms isolated in different sites of infections and bloodstream

Micro-organism	Site of infection	Blood^a^
*Escherichia coli*	54 (35.3)	27 (43.5)
*Streptococcus pneumoniae*	23 (15)	7 (11.3)
*Streptococcus *spp.	15 (9.8)	8 (5.2)
*Klebsiella *spp.	12 (7.8)	6 (9.7)
*Proteus mirabilis*	7 (4.6)	2 (3.2)
*Legionella pneumophila*	7 (4.6)	-
*Enterococcus *spp.	6 (3.9)	1 (1.6)
*Pseudomonas *spp	5 (3.3)	-
*Staphylococcus aureus*	4 (2.6)	4 (6.4)
*Neisseria meningitidis*	4 (2.6)	3 (4.8)
*Bacteroides fragilis*	4 (2.6)	2 (3.2)
*Chlamydia pneumoniae*	3 (1.9)	-
*Salmonella enteritidis*	2 (1.3)	1 (1.6)
*Mycoplasma pneumoniae*	2 (1.3)	
*Prevotella oris*		1 (1.6)
*Aeromonas hydrophila*		1 (1.6)
*Enterobacter *spp.	1 (0.65)	
*Nocardia asteroids*	1 (0.65)	
*Staphylococcus *spp.	1 (0.65)	
*Leptospira*	1 (0.65)	
*Coxiella burnetii*	1 (0.65)	
Total	153 (100)	63 (100)

Values are expressed as *n *(%). ^a^Six episodes of polymicrobial bacteraemia were detected.

**Table 4:** Bivariate analysis of risk factors for in-hospital mortality

Factor	Nonsurvivors (*n *= 52)	Survivors (*n *= 172)	RR (95% CI)	P
Sex			0.46 (0.28–0.74)	0.007
Male	21 (40.4)	106 (61.6)		
Female	31 (59.6)	66 (38.4)		
Age (years)^a^	61.5 (47.2–71.7)	67 (56.5–75.7)		0.09
Hepatic cirrhosis	5 (9.6)	9 (5.2)	1.93 (0.48–6.75)	0.32
Immunosuppression	5 (9.6)	4 (2.3)	4.47 (0.91–23.25)	0.03
COPD	5 (9.6)	17 (9.9)	0.97 (0.27–2.93)	0.95
End-stage renal disease	5 (9.6)	7 (4.1)	2.51 (0.6–9.62)	0.15
Chronic cardiac failure	2 (3.8)	3 (1.7)	2.25 (0.18–20.16)	0.33
Diabetes mellitus	13 (25)	44 (25.6)	0.97 (0.45–2.09)	0.93
Noncured malignancy	8 (15.4)	4 (2.3)	1.92 (7.64–35.84)	0.001
Alcoholism	5(9.6)	18 (10.5)	0.91 (0.25–2.73)	0.86
Smoking habit	8 (15.4)	41 (23.8)	0.65 (0.33–1.29)	0.25
APACHE II^a^	18.5 (14–23.75)	12 (8–17)		<0.001
APACHE II admission ICU^a,b^	22.5 (18–26)	15.5 (10–19.75)		<0.001
SOFA (1)^a,c^	6 (3–11)	3 (1–7)		<0.001
SOFA admission ICU^a,b^	10.5 (6.75–14)	6 (3–9.25)		<0.001
Bacteraemia	13 (25)	43 (25)	1 (0.46–2.16)	1
Site of infection				
Urologic	4 (7.7)	30 (17.5)	0.39 (0.1–1.21)	
Central nervous system	3 (5.7)	10 (5.8)	0.75 (0.13–2.84)	
Other	0 (0)	4 (2.3)	0 (0–5.04)	
Soft tissue	3 (5.8)	14 (8.1)	0.69 (0.12–2.63)	
Abdomen	23 (44.2)	59 (34.3)	1.52 (0.77–2.99)	
Unknown	4 (7.7)	5 (2.9)	2.78 (0.53–13.42)	
Lung	15 (28.8)	50 (29.1)	0.67 (0.32–1.37)	
Genotype				
-308 TNF-α promoter polymorphism			1.28 (0.49–3.46)	0.59
GG	45 (86.5)	141 (82%)		
GA/AA	7 (13.5)	31 (18)		
TNF-β (*Nco*I polymorphism)			0.74 (0.37–1.5)	0.37
GG/GA	17 (33.7)	68 (39.6)		
AA	35 (67.3)	104 (60.4)		
IL-10-1082			0.7 (0.22–1.88)	0.42
GG	6 (11.6)	27 (15.7)		
GA/AA	46 (88.4)	145 (84.3)		
Genotype -308 TNF GA/AA, TNF-β AA, IL-10-1082 GG			0.92 (0.45–1.84)	0.8
Yes	18 (34.1)	63 (36.6)		
No	34 (65.4)	109 (63.4)		
Empirical antibiotic therapy				<0.001
Adequate	24 (46.1)	118 (68.6)	0.39 (0.20–0.77)	
Inadequate	12 (23.1)	4 (2.3)	12.6 (3.53–55.57)	
Not evaluable	16 (30.8)	50 (29.1)	1.08 (0.52–2.24)	
Delayed surgical intervention^a,d^	16 (7–36)	12.5 (8–24)		0.77
Delayed of AAT^a^	7 (4–28)	5 (3–10)		0.008

Unless otherwise stated, values are expressed as *n *(%). ^a^Results expressed as median (25th to 75th percentiles). ^b^Only 178 patients were admitted to the ICU. ^c ^SOFA (1) means SOFA score in the first 24 hours in the hospital. ^d^Only in 'surgical patients' (*n *= 93). AAT, appropriate antibiotic therapy; APACHE, Acute Physiology and Chronic Health Evaluation; CI, confidence interval; COPD, chronic obstructive pulmonary disease; ICU, intensive care unit; RR, relative risk; SOFA, Sequential Organ Failure Assessment.

**Table 5:** Bivariate analysis of risk factors for in-hospital mortality in patients with septic shock

Factor	Nonsurvivors (*n *= 49)	Survivors (*n *= 65)	RR (95% CI)	P
Sex				0.07
Male	18 (36.7)	35 (43.9)		
Female	31 (63.3)	30 (46.1)		
Age (years)^a^	62.5 (55–75)	60 (45–72)		0.11
Hepatic cirrhosis	5 (10.2)	4 (6.2)	1.73 (0.35–9.21)	0.32
Immunosuppression	4 (8.2)	2 (3.1)	2.8 (0.38–31.92)	0.21
COPD	5 (10.2)	5 (7.7)	1.36 (0.29–6.30)	0.42
End-stage renal disease	5 (10.2)	3 (4.6)	2.35 (0.43–15.79)	0.21
Chronic cardiac failure	2 (4.1)	2 (3.1)	1.34 (0.09–19.07)	0.58
Diabetes mellitus	13 (26.5)	18 (27.7)	0.94 (0.38–2.35)	0.89
Noncured malignancy	6 (12.2)	1 (1.5)	8.9 (1.01–417)	0.24
Alcoholism	5 (10.2)	6 (9.2)	1.12 (0.25–4.71)	0.55
Smoking habit	8 (16.4)	10 15.4)	1.07 (0.35–3.29)	0.89
APACHE II^a^	18 (14–22)	17 (13–21)		0.09
SOFA (1)^a,b^	6.5 (3–11)	8 (3–11)		0.8
Bacteraemia	13 (26.5)	26 (40)	0.54 (0.22–1.30)	0.2
Genotype				
-308 TNF-α promoter polymorphism			1.80 (0.61–5.43)	0.42
GG	42 (85.7)	50 (77)		
GA/AA	7 (14.3)	15 (23)		
TNF-β (NcoI polymorphism)			0.66 (0.26–1.39)	0.19
GG/GA	16 (32.6)	29 (44.6)		
AA	33 (67.4)	36 (55.4)		
IL-10-1082			0.62 (0.18–1.96)	0.89
GG	6 (12.2)	12		
GA/AA	43 (81.8)	53		
Genotype TNF -308 GA/AA, TNF-β AA, IL-10-1082 GG			1.09 (0.46–2.61)	0.65
Yes	16 (32.6)	20		
No	33 (67.4)	45		
Delayed AAT^a^	7.5 (4–28.5)	5.5 (3–12)		0.03

Unless otherwise stated, values are expressed as *n *(%). ^a^Results expressed as median (25th to 75th percentiles). ^b^SOFA (1) means SOFA score of the first 24 hours in the hospital. AAT, appropriate antibiotic therapy; APACHE, Acute Physiology and Chronic Health Evaluation; CI, confidence interval; COPD, chronic obstructive pulmonary disease; ICU, intensive care unit; RR, relative risk; SOFA, Sequential Organ Failure Assessment.

**Table 6:** Bivariate analysis of risk factors for impairment of the inflammatory response

Factor	Impairment of the inflammatory response			*P*
		
	Yes (*n *= 57)	No (*n *= 106)	RR (95%CI)	
Sex			0.39 (0.19–0.79)	0.004
Male	25 (46.3)	71 (67)		
Female	32 (53.7)	35 (33)		
Age^a^	60 (50–72)	61 (48–70.2)		0.5
Hepatic cirrhosis	8 (14)	3 (2.8)	5.61 (1.26–33.83)	0.01
Immunosuppression	3 (5.3)	3 (2.8)	1.91 (0.25–14.67)	0.42
COPD	3 (5.3)	12 (11.3)	0.44 (0.1–1.72)	0.2
End-stage renal disease	3 (5.3)	4 (3.8)	1.42 (0.2–8.7)	0.7
Chronic cardiac failure	2 (3.5)	1 (0.94)	3.82 (0.2–227)	0.28
Diabetes mellitus	12 (21)	26 (24.5)	0.82 (0.35–1.9)	0.61
Noncured malignancy	4 (7)	4 (3.8)	1.92 (0.34–10.7)	0.71
Alcoholism	7 (12.3)	12 (11.3)	1.21 (0.4–3.58)	0.85
Smoking habit	10 (17.5)	30 (28.3)	0.22 (0.54–1.28)	0.12
APACHE II^a^	14 (10–16.5)	10 (6–14)		<0.0001
SOFA (1)^a,b^	3 (2–6)	2 (1–4)		0.003
Bacteraemia	18 (31.6)	14 (13.2)	3.03 (1.28–7.22)	0.005
Genotype				
-308 TNF-α promoter polymorphism			0.88 (0.33–2.37)	0.77
GG	48 (84.2)	91 (85.8)		
GA/AA	9 (15.8)	15 (14.2)		
TNF-β (*Nco*I polymorphism)			1.25 (0.61–2.54)	0.5
GG/GA	24 (42.1)	39 (28.3)		
AA	33 (57.9)	67 (63.2)		
IL-10-1082			1.09 (0.46–2.61)	0.8
GG	8 (14)	15 (14.2)		
GA/AA	49 (86)	91 (85.8)		
Genotype -308 TNF GA/AA, TNF-β AA, IL-10-1082 GG			1.48 (0.71–3.09)	0.26
Yes	18 (31.5)	43 (40.6)		
No	39 (68.5)	63 (59.4)		
Delayed AAT^a^	10 (6–25)	5 (2.5–8)		<0.001

Unless otherwise stated, values are expressed as *n *(%). ^a^Results expressed as median (25th to 75th percentiles). ^b^SOFA (1) means SOFA score of the first 24 hours in the hospital. AAT, appropriate antibiotic therapy; APACHE, Acute Physiology and Chronic Health Evaluation; CI, confidence interval; COPD, chronic obstructive pulmonary disease; ICU, intensive care unit; RR, relative risk; SOFA, Sequential Organ Failure Assessment.
